# Supplementary figures and images for: Phagocytes produce prostaglandin E2 in response to cytosolic Listeria monocytogenes
Source: PLoS Pathog. 2021 Sep 23;17(9):e1009493. doi: 10.1371/journal.ppat.1009493 (PMC8491950; doi:10.1371/journal.ppat.1009493)

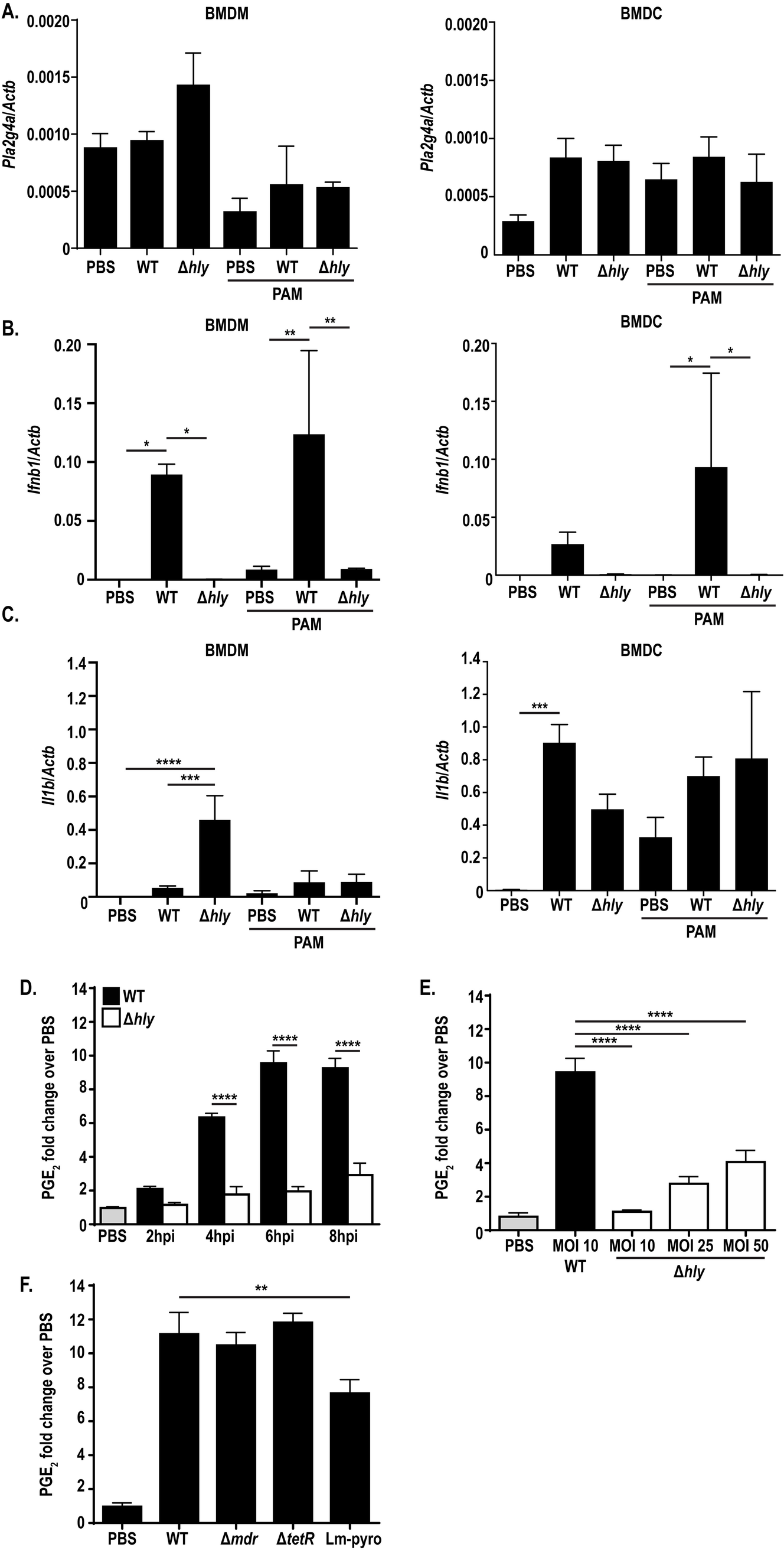

Supplement: S1 Fig — Activation of type I IFN or inflammasomes does not substantially alter PGE2production. BMDMs or BMDCs were infected with the indicated strains of L. monocytogenes at an MOI 10 +/- the TLR2 agonist PAM3CSK4 and assessed 6hpi for the expression of Pla2g4a (encoding cPLA2, A), Ifnb1 (encoding interferon β, B), and Il1b (encoding IL-1β, C) by qRT PCR. PAM3CSK4-primed BMDMs were infected at an MOI 10 for six hours (unless otherwise specified) with the indicated strains of L. monocytogenes. PGE2 levels in the supernatant was then assessed by ELISA (D-F). Data are a combination of three independent experiments. Significance was determined by a one-way ANOVA with Bonferroni’s correction. *p < 0.05, **p < 0.01, ***p < 0.001, ****p < 0.0001. (TIF) [file ppat.1009493.s001.tif]

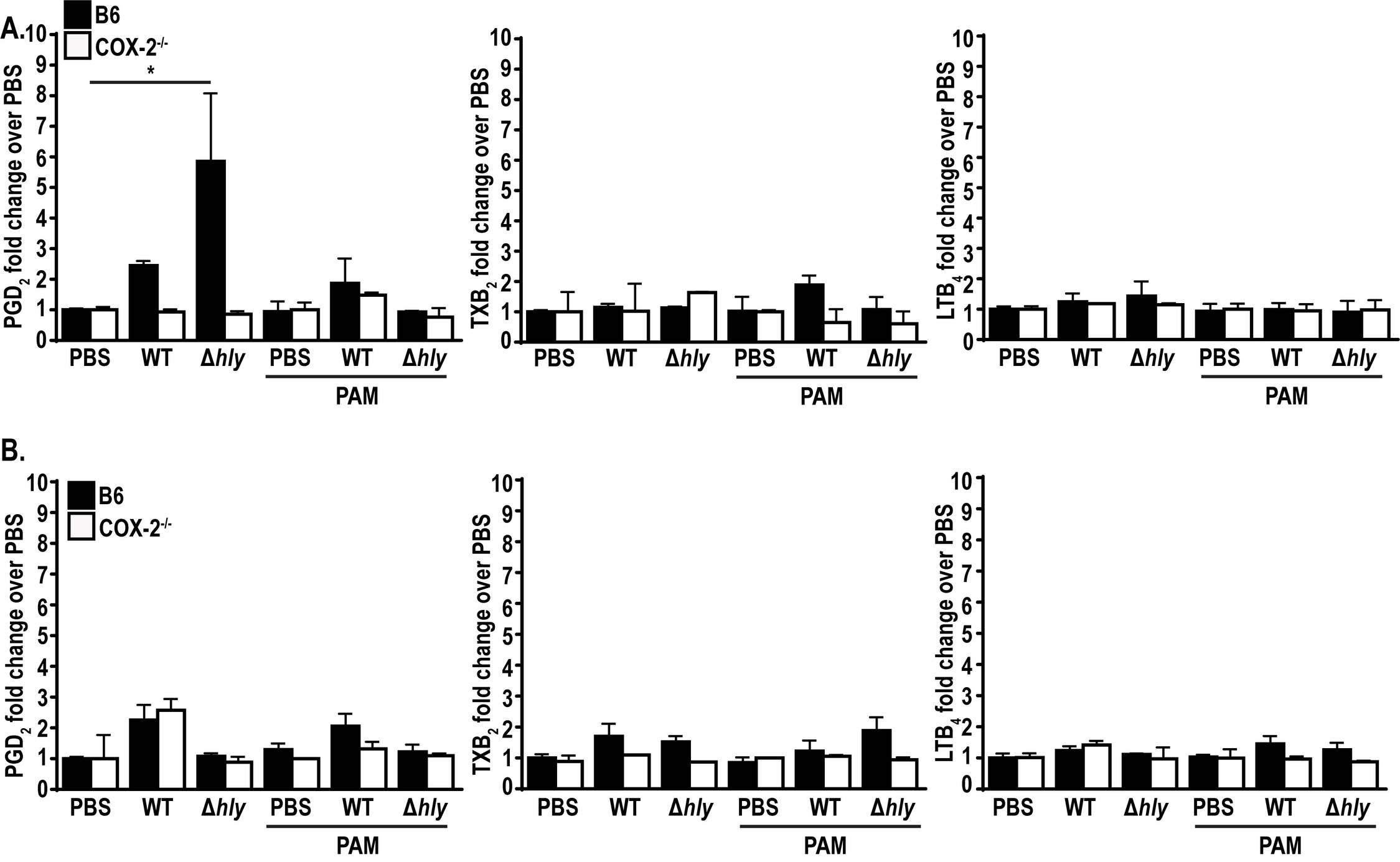

Supplement: S2 Fig — Wild-type or COX-2-/- BMDMs (A) or BMDCs (B) were infected with the indicated strains of L. monocytogenes at an MOI of 10 +/- the TLR2 agonist PAM3CSK4. Supernatant was harvested 6hpi and assessed for prostaglandin D2 (PGD2), thromboxane B2 (TXB2), or leukotriene B4 (LTB4). Data was normalized to d-PGE2 and fold change is relative to PBS treated controls. Data are a combination of two independent experiments. Significance was determined by a one-way ANOVA with Bonferroni’s correction. *p < 0.05. (TIF) [file ppat.1009493.s002.tif]

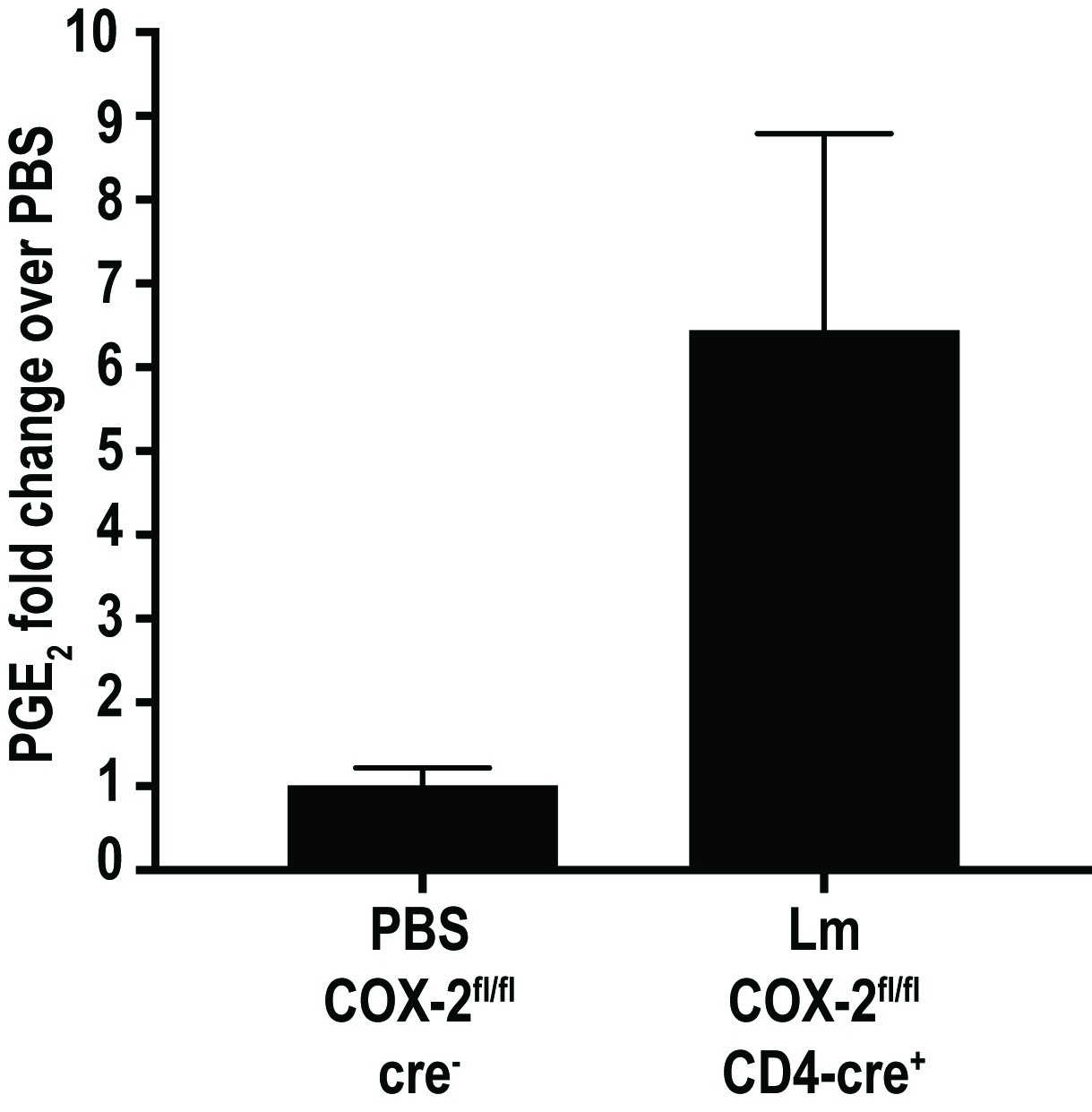

Supplement: S3 Fig — Indicated strains of mice were immunized with 107 LADD L. monocytogenes or PBS control. 12hpi spleens were harvested and assessed for PGE2 by mass spectrometry. Data was normalized to d-PGE2 levels and fold change is compared to PBS controls. Data are representative of two independent experiments. Significance was determined by a one-way ANOVA with Bonferroni’s correction. (TIF) [file ppat.1009493.s003.tif]

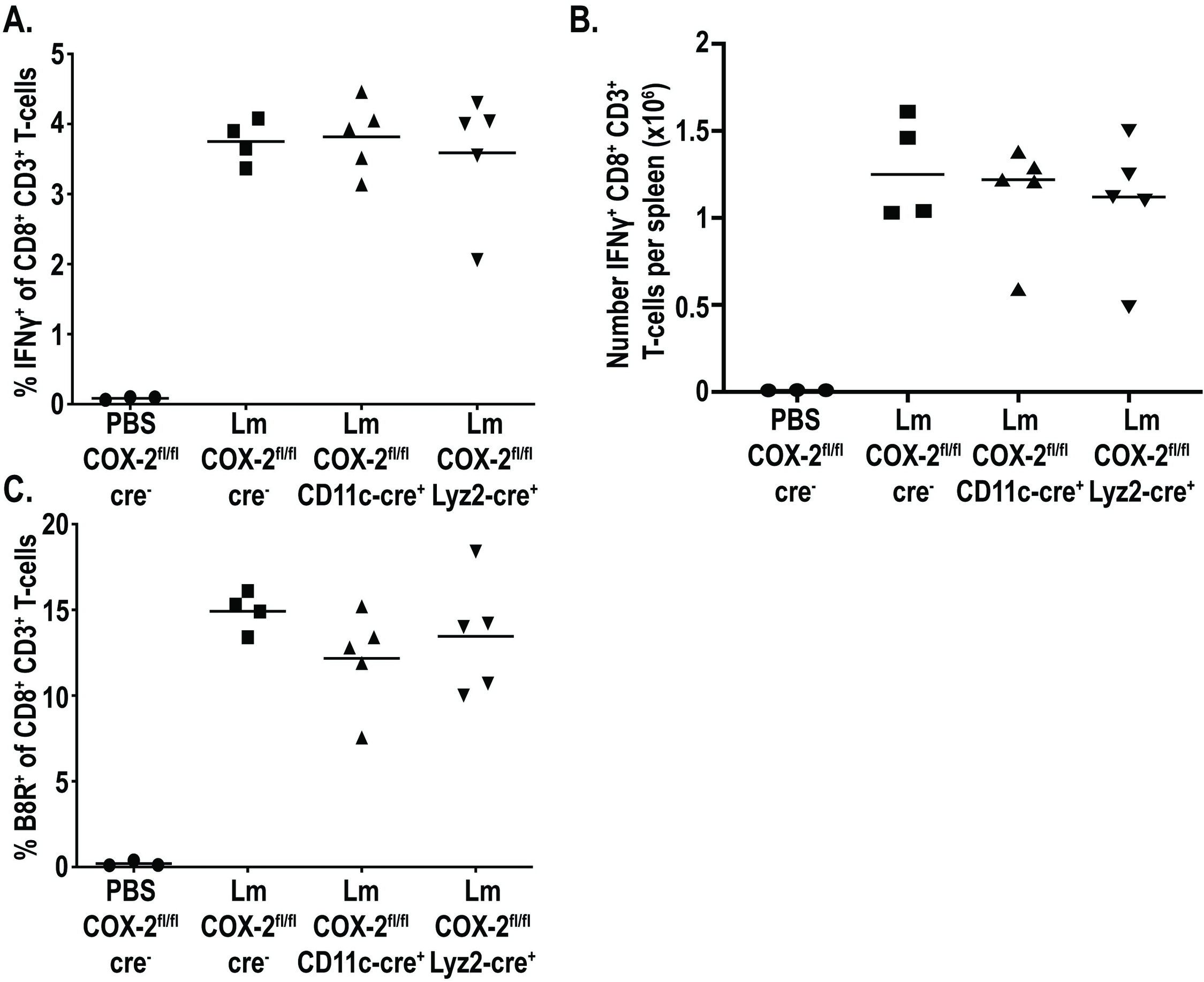

Supplement: S4 Fig — Indicated strains of mice were infected with 107 LADD L. monocytogenes. 7dpi splenocytes were examined for OVA-specific CD8+ T-cell responses. %IFNγ (A) or number IFNγ (B) per spleen was assessed. B8R-tetramer positive CD8+ T-cell responses were also assessed (C). Data are a representative of two independent experiments of 4–5 mice per group. Significance was determined by a Mann-Whitney U test. (TIF) [file ppat.1009493.s004.tif]

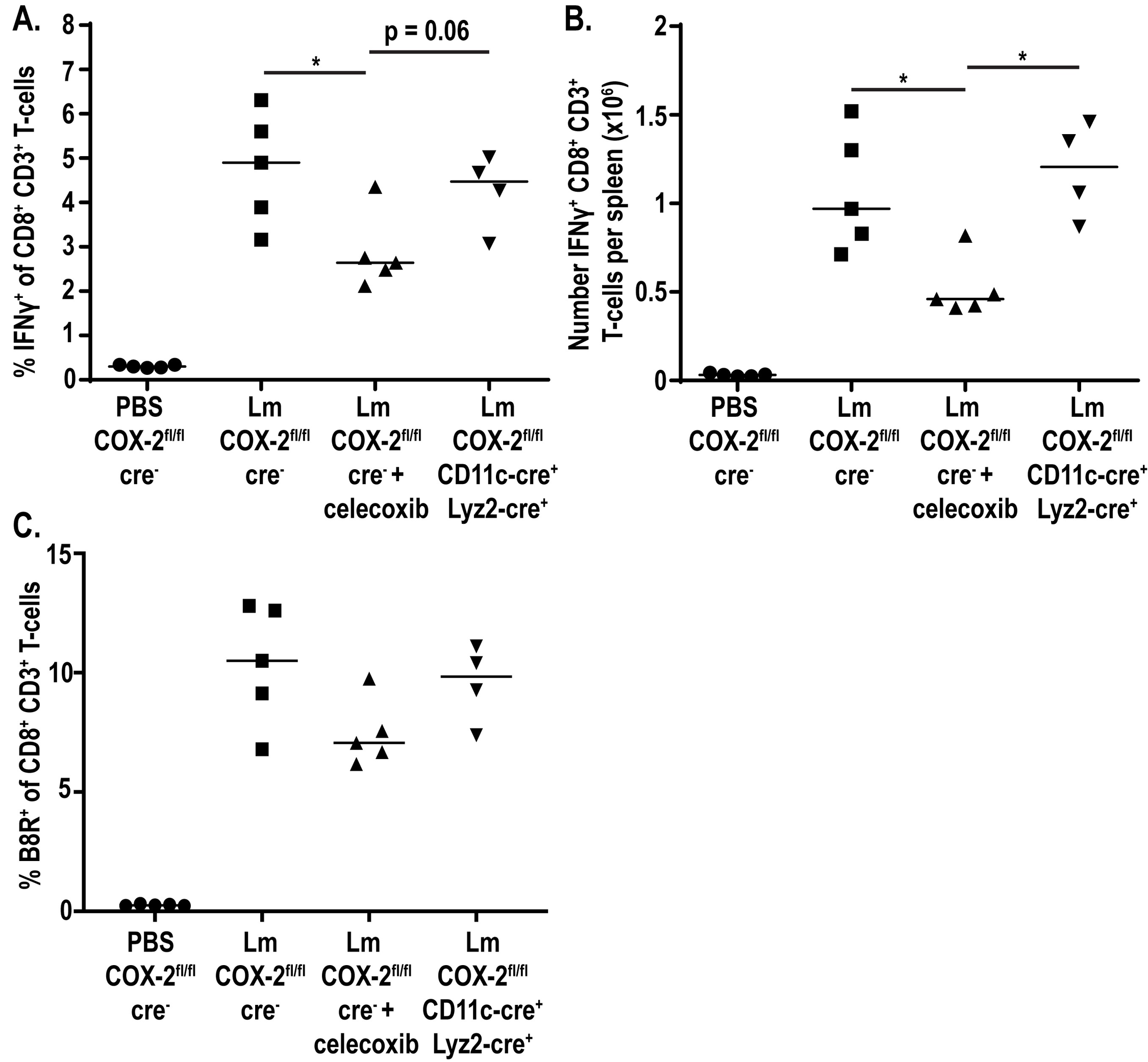

Supplement: S5 Fig — Indicated strains of mice were infected with 107 LADD L. monocytogenes. 7dpi splenocytes were examined for OVA-specific CD8+ T-cell responses. %IFNγ (A) or number IFNγ (B) per spleen was assessed. B8R-tetramer positive CD8+ T-cell responses were also assessed (C). Data shown are representative of two independent experiments of 3–5 mice per group. Significance was determined by a Mann-Whitney U test (B-C). *p < 0.05. (TIF) [file ppat.1009493.s005.tif]

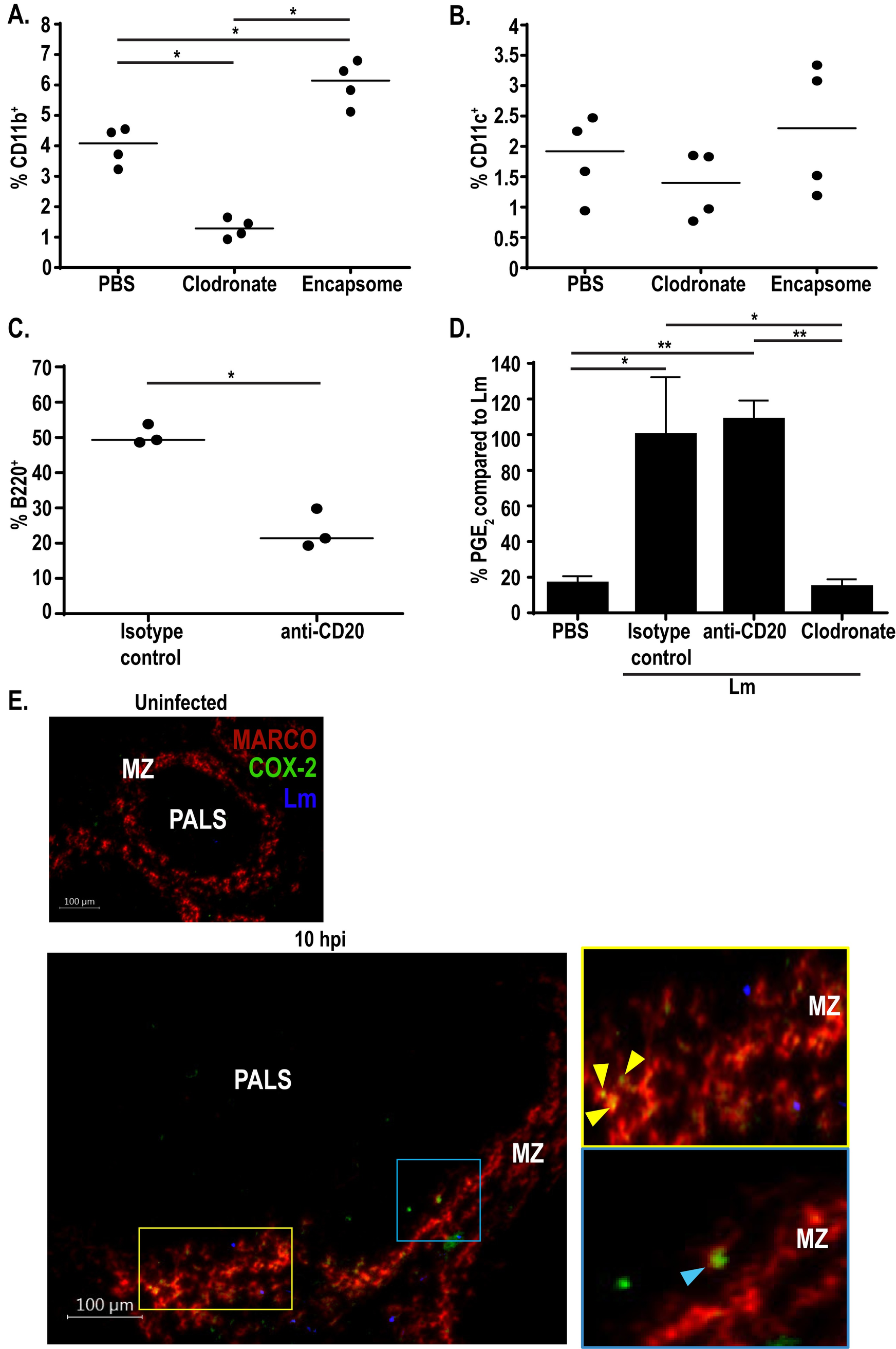

Supplement: S6 Fig — Anti-CD20 treatment depletes splenic B220+cells, but does not influence PGE2production. C57BL/6 mice were dosed with 200μL clodronate, liposome control (encapsome), or PBS 24 hours prior to immunization with 107 LADD L. monocytogenes. 12hpi spleens were harvested and assessed for CD11b+ (A) and CD11c+ (B) populations by flow cytometry. C57BL/6 mice were dosed with 50μg anti-CD20 or isotype control 24 hours prior to immunization with 107 LADD L. monocytogenes. 12hpi spleens were harvested and assessed for B220+ populations by flow cytometry (C) or PGE2 levels by mass spectrometry (D). COX-2fl/fl CD11c-cre+ Lyz2-cre+ were infected with 107 LADD L. monocytogenes. 10hpi spleens were harvested, cryosections were cut, and sections were stained for L. monocytogenes, COX-2, and MARCO (E). Data shown are a combination of two independent experiments. Significance was determined by a Mann-Whitney U test. *p < 0.05, **p < 0.01. (TIF) [file ppat.1009493.s006.tif]
