# Supplementary material for: Phagocytes produce prostaglandin E2 in response to cytosolic Listeria monocytogenes
Source: PLoS Pathog. 2021 Sep 23;17(9):e1009493. doi: 10.1371/journal.ppat.1009493 (PMC8491950; doi:10.1371/journal.ppat.1009493)
Supplement: Attachment — Submitted filename: Response to Reviewers.pdf [file ppat.1009493.s007.pdf]

We thank the reviewers for their thoughtful critiques. We have responded to the reviewer concerns and have added seven new figure panels as well as modifications to the title and text of the manuscript. Responses to these reviewer comments have resulted in a more complete manuscript. Our responses to the reviewer comments are as follows:

Reviewer #1: 1. My major criticism involves the in vitro analysis using cultured macrophages and DCs and their conclusion that PGE2 production is a result of Lm entering the cytosol. Host cells were infected with an equal amount of WT or LLO-minus Lm and PGE2 levels were evaluated after 6 hours. At this time point, there would be approximately 50-times more WT Lm than LLO-minus.

1a. I suggest that the authors show a time-course and identify the time of maximum PGE2.

We thank the reviewer for this suggestion and have provided a time-course of PGE_2 production. (Supplemental figure 1D). Our data suggest that a small amount of PGE_2 is produced as early as 2hpi during wild-type *L. monocytogenes* infection. PGE_2 levels peak around 6hpi and is maintained until at least 8hpi.

1b. They should show the levels of PGE2 if more LLO-minus are added to be equivalent to the numbers of WT.

We agree that differential burden between the two strains could influence PGE_2 levels. We repeated our assessment of PGE_2 production *ex vivo* in response to increasing MOIs of Δ*hly L. monocytogenes*. Even at an MOI of 50, Δ*hly L. monocytogenes* induced significantly less PGE_2 than wild-type *L. monocytogenes* infected at an MOI of 10 (Supplemental figure 1E). Furthermore, when we assessed PGE_2 levels at earlier timepoints when the bacterial numbers of the two strains are more equivalent, only wild-type and not Δ*hly L. monocytogenes* induced PGE_2 (Supplemental figure 1D). These data are consistent with our *in vivo* data highlighting that despite equivalent burdens, Δ*hly L. monocytogenes* infection fails to induce PGE_2 production. Taken together, we feel that these new data in addition to the previously included *in vivo* data suggest that differences in PGE_2 are not only due to decreased bacterial burdens of the Δ*hly L. monocytogenes*.

1c. Since PGE2 has a very short half-life, it is possible that PGE2 is induced early but is absent at the 6h time course.

We agree with the reviewer that PGE_2 is labile and earlier peaks of production could be missed. However, in our mass spectrometry analysis we not only assess levels of PGE_2, but also identify stable byproducts. At the six-hour time point assessed, we do not detect any of the downstream byproducts, suggesting that we have not missed an earlier PGE_2 peak. Nevertheless, we included both wild-type and the Δ*hly* mutant in the time-course suggested in comment 1a to assess PGE_2 kinetics for both strains. We did not see any PGE_2 production at any time-point analyzed with the Δ*hly* mutant (Supplemental figure 1D). This suggests that infection of Δ*hly L. monocytogenes* does not simply alter the kinetics of PGE_2 production leading to an earlier peak.

1d. I am curious if they need to use mass spec for their analysis or would using commercially available ELISAs make this analysis easier?

Use of ELISAs is a good suggestion and certainly would make some of these experiments easier. As such, we performed the new experiments included as supplemental figures 1D-F by ELISA and the data match quite consistently with the data in Fig. 1D done by mass spectrometry. We chose to perform the original experiments in the manuscript by mass spectrometry for a few reasons. First, the available ELISA kits do not perform as well *in vivo* and so for consistency we chose to analyze both our *in vivo* and *ex vivo* experiments by mass spectrometry. Secondly, as briefly mentioned in the response to 1C, use of mass spectrometry not only allows us to look for PGE_2, but also stable byproducts of PGE_2. This gives us confidence that a lack of PGE_2 is not simply due to degradation or further metabolism. Additionally, it allows us to assess levels of other eicosanoids beyond PGE_2 to allow for a more holistic understanding of lipid changes during infection.

1e. In the discussion, the authors mention STING, inflammasomes and type I IFN. It would be nice to see PGE2 production in mice lacking all of these.

We agree with the reviewer that this is a very intriguing question, particularly with past literature identifying crosstalk between these pathways. To address this comment, we have provided analysis of PGE_2 levels during *ex vivo* infection with strains of *L. monocytogenes* that differentially activate these pathways. During infection with strains that alter type I IFN (Δ*tetR* --hyperinduction of IFNβ, Δ*mdr*-hypoinduction of IFNβ), there was no change in PGE_2 production (Supplemental figure 1F). Infection with Lm-pyro, a strain that hyperactivates the inflammasome, surprisingly led to a slight but significant decrease in PGE_2 production (Supplemental figure 1F). This suggests that there may be some crosstalk between inflammasomes and PGE_2 production. As inflammasome activation leads to pyroptosis, this decrease in production may alternatively be driven by lower viable cell number. Additionally, our lab is currently working on a manuscript focused on T-cell priming in mice deficient in caspase-1/11, type I IFN receptor (IFNAR), or in a novel double caspase-1/11 and IFNAR-deficient mouse. A portion of this manuscript is focused on understanding changes in eicosanoids in the absence of one or both of these pathways. Due to the lack of robust changes *ex vivo* as well as a deeper investigation of eicosanoids in this additional manuscript, we feel that mass spectrometry analysis of PGE_2 *in vivo* is out of the scope of this current manuscript.

1f. As the authors point out, PGE2 production involves calcium signaling and they suggest that LLO pore formation may be involved in triggering PGE2. They also mention a strain of Lm that it would be interesting if a strain engineered to delete LLO in the cytosol was evaluated (ref 51). I suggest they try this strain in vitro and possible in vivo as well.

We agree that this is an interesting model to assess the role of LLO pore formation in driving PGE_2 production. The Portnoy lab generously shared this strain and we have subsequently assessed PGE_2 levels once *ex vivo* and *in vivo.* In both models, we detected similar PGE_2 production after infection compared to infection with wild-type *L. monocytogenes.* Our concern with this strain is that currently the kinetics of *hly* gene excision are unknown. Because

of this, we cannot be confident that the excision is quick enough to prevent LLO-mediated membrane pore formation. We have included these data below for your reference and can include it in the manuscript, however, with the caveat mentioned above we are concerned that the data may not fully reflect a complete loss of LLO-driven calcium influx and until such a time as these kinetics can be determined we believe the data should not be published. We are currently working toward this goal.

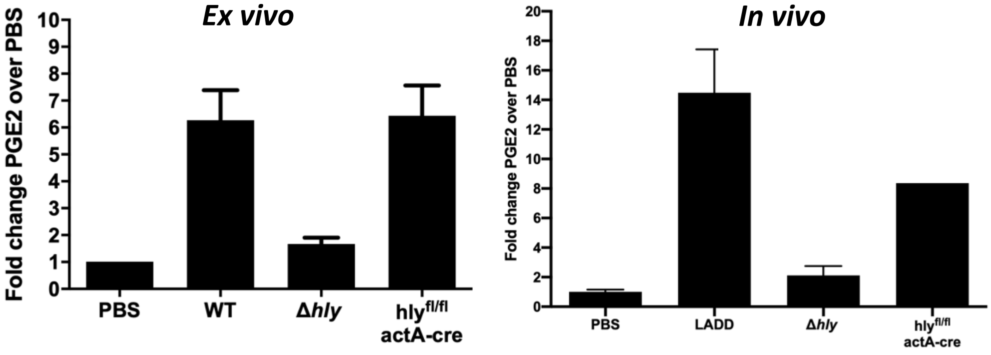

2. the current study strongly suggest that PGE2 is induced by cytosolic access by Lm in phagocytes, leading to optimal CD8+ T-cell responses. However, although the authors demonstrate that cytosolic Lm induces PGE2 ex vivo and in vivo, the data showing that this process necessarily occurs in phagocytes for optimal T-cell priming is lacking. Part of challenge is, as the authors mention, that Clodronate treatment removes antigen presenting cells, making analysis of CD8+ T-cell priming irrelevant under those circumstances. As a result, the required experiments are not yet done, and would require mice lacking complete PGE2 induction in the MZMs. As an alternative, the authors look at MZMs as the possible source of PGE2 during wt Lm infection using IHC for colocalization studies (Fig5 c-d). While wt Lm does co-localize with MARCO and COX-2, the in vitro data demonstrate that both WT and LLO-minus Lm induce COX-2(fig 1C). Nevertheless, based on the current data and the previous literature, the results are highly suggestive that PGE2 is induced by cytosolic bacteria in phagocytes for optimal T-cell priming. However, this has not been completely demonstrated due to the removal of APCs by chlodronate, resulting in key CD8 + T-cell priming assays not being shown. The COX2 pathway in MZMs should be removed specifically, and T cells assayed after immunization.

We appreciate the point made by this reviewer, and we agree that deletion of COX-2 in MZMs would further support our hypothesis. However, as we have demonstrated that even a small amount of PGE_2 is sufficient for generating a T-cell response, deletion of COX-2 in MZMs individually would likely not impact cell-mediated immunity. In order to impact $CD8^+$ T-cell responses, we would have to eliminate COX-2 in all relevant phagocytic cell populations that contribute to PGE_2 during *L. monocytogenes* infection, requiring generation of at least quadruple transgenic mice (COX-$2^{fl/fl}$, CD11c-cre^+, Lyz2-cre^+, SIGN-R1-cre^+). Particularly because the SIGN-R1-cre mouse is not commercially available, this would require a large amount of time and resources and we therefore believe this is beyond the scope of this manuscript. Nevertheless, to address this reviewer's concern we have assessed COX-2 expression in our mice deficient in COX-2 in both CD11c and Lyz2 cells (COX-$2^{fl/fl}$ CD11c-cre^+ Lyz2-cre^+) by

immunohistochemistry and have found that in these mice the remaining COX-2 expression is strikingly located in the marginal zone and colocalizes with MZMs (Supplemental figure 6E), supporting our hypothesis that MZMs may be contributing to the residual PGE_2 production. Additionally, we have substantially softened our language that phagocyte-derived PGE_2 specifically is driving T-cell responses at multiple places throughout the manuscript (eg. changed title, deleted or changed text at lines 24, 33, 43, 51, 125, 451 just to highlight a few).

3. To strengthen the paper, the authors should also bolster the concept that only the cytosolic strain induces PGE2 in MZMs, by comparing the LLO-minus and wt strains using the IHC methods already employed, to show that only cytosolic bacteria co-localize with MARCO and COX-2, whereas LLO-minus strains should not co-localize, providing further evidence that only cytosolic bacteria induce the PGE2 pathway in vivo. This work should not be too difficult or take too long to do since similar experiments were done already. The paper would significantly benefit from this figure since it is key to the main concept that LLO and wt induce PGE2 differentially in MZMs.

Our data presented in Figure 1 suggests that modulation of PGE_2 production (particularly in macrophages) is driven by mechanisms outside of protein expression. We found by western blot (Figure 1C) that infection of Δ*hly L. monocytogenes* still leads to some expression of COX-2, despite inducing lower levels of PGE_2. Due to these data, we suspect that PGE_2 production is additionally regulated through post translational mechanisms rather than at the level of translation (see response to reviewer 3, comment 1). We expect that Δ*hly L. monocytogenes* would similarly induce COX-2 protein expression *in vivo.* For this reason, we do not believe additional immunohistochemistry using the Δ*hly* strain would be informative in this instance and may be confounding.

Reviewer #2: The authors outline in the Discussion a number of possible experiments that could move the studies forward and a variety of different directions. Given the paper's title and its focus on cytosolic induction of PGE2 synthesis by phagocytes, my opinion is that the authors should minimally seek to address the following:

(1) Provide additional data to more directly support that conclusion that phagocytes are an essential source of PGE2 production. Chlodronate depletion was shown to suppress measured PGE2 production, but in this is a blunt tool. It remains possible that such treatment suppresses PGE2 by non-phagocytes, e.g. by triggering massive induction of cell death? Can the authors demonstrate that depleting another cell population through a process that stimulates cell death (e.g. depletion of B cells with anti-CD20 or using a DTR/DT system) fails to eliminate measurable PGE2 in B6 or Cox-2FL/FL x Lyz2-cre + Cd11c-cre mice?

We thank the reviewer for this suggestion and agree that large amounts of cell death may suppress PGE_2 production. We performed a B cell depletion using anti-CD20 as suggested by this reviewer. As discussed in the text (lines 391-398), we dosed mice with anti-CD20 twenty-four hours prior to immunization with *L. monocytogenes*, leading to a depletion of 50% of the B cells in the spleen (supplementary figure 6C). As B cells represent approximately half of the cells in the spleen, a 50% B cell depletion results in a massive amount of cell death. The amount of

cell death induced was likely larger than during clodronate treatment, as phagocytic cells comprise a much smaller portion of the spleen (5-10%). We found that treatment of mice with anti-CD20 did not reduce PGE_2 production after infection compared to mice treated with an isotype control (supplemental figure 6D). This suggests that the cell death induced by clodronate treatment was not the major cause of abrogated PGE_2 production.

(2) Provide evidence that phagocyte PGE2 is critical for initiating the CD8+ T cell response. The lack of a reduction in CD8+ T cells in the Cox-2FL/FL x Lyz2-cre + Cd11c-cre mice could, as the authors propose, implicate that critical PGE2 can also be released by MZ macrophages. However, it is also possible that PGE2 from some other cell type is sufficient. Since chlodronate would be expected to also deplete DC, the best way to address this would be to crossing the floxed Cox2 allele onto a new cre driver that targets other phagocytes (and possibly crossing these mice again to the existing strain that combines Lyz2-cre and Cd11c-cre). The authors mention SIGNR1-cre in the Discussion. Mice that delete COX2 more broadly in macrophages could also be used (e.g. Csf1r-cre or Cd11b-cre). The authors might further consider using a cre driver that targets all hematopoietic cells to confirm that immune cells are key for PGE2 production that leads to the CD8+ T cell activation.

We agree that further generation of new transgenic mice would help solidify our hypothesis. Unfortunately cre-drivers such as CD11b also do not effectively target MZMs (Abram et al., J Immunol Methods, 2014), and hematopoietic cre drivers would not enable analysis of PGE_2 derived from phagocytic populations specifically. To our knowledge, the efficacy of Csf1r-cre specifically on MZMs has not been examined, though development of a subset of $MARCO^+$ MZMs is known to be independent of M-CSF, suggesting that this cre-driver may also lack efficacy in these cells (Ito et al., Arch Histol Cytol., 1999). Furthermore, as multiple phagocytic populations produce PGE_2, to test the hypothesis that PGE_2 derived from multiple phagocytic populations is important for T-cell responses a transgenic deleting COX-2 in all the relevant subsets would be required (see response to Reviewer 1, major comment 2). We believe that generation of these at least quadruple transgenic mice is out of the scope of the current manuscript. However, we have softened the language (changed title, deleted or changed text at lines 24, 33, 43, 51, 125, 451 just to highlight a few) and included an analysis of COX-2 expression in our mice deficient in COX-2 in both $Lyz2^+$ and $CD11c^+$ cells and found that while the vast majority of COX-2 expression was gone in the double transgenics, the remaining COX-2 expression was located in the MZ (Supplemental figure 6E, see response to Reviewer 1, major comment 2).

(3) Provide additional evidence to support the notion that PGE2 production requires access of L. monocytogenes to the macrophage cytosol. Although the delta-HLY strain did not trigger PGE2 in vivo, is seems that this strain was capable of triggering some PGE2 production in cultured BMDMs. This raises the possibility that the induction of PGE2 is not dependent on any specific cytosol detection mechanism and further that the lack of T cell priming by the delta-HLY strain could be due to some other defect. Indeed, HLY has been ascribed a number of functions in addition to promoting release of L. monocytogenes

from vacuoles. It is also possible that elevated PGD2 or some other factor produced selectively during infection by the delta-HLY strain is responsible for the impaired T cell response. The authors should indirectly address this question by demonstrating if the triggering of PGE2 production by WT L. monocytogenes in vitro (and in vivo?) is dependent (or not) on inflammasome activation by the WT (but not delta-HLY) bacteria. It would also be useful to include experiments mentioned in the discussion using bacterial strains which have reduced ability to trigger inflammasome activation to see if these also have reduced ability to stimulate PGE2 and T cell responses. Even if these results show that the inflammasome is not required, they will provide useful information and advance the impact of the paper. Finally, important controls missing here include confirmation that a delta-HLY strain expressing OVA or B8R fails to activate the respective T cells and, assuming not, to show if the presence of the mutant strain does or does not suppress PGE2 production and T cell priming in mice that are also infected with WT bacteria.

Previous literature has conclusively demonstrated that Δ*hly L. monocytogenes* fails to induce robust $CD8^+$ T-cell priming (Brunt et al., J Immunol. 1990; Bahjat et al., Infect. Immun., 2006). As requested by this reviewer, we have repeated this analysis assessing $CD8^+$ T-cell responses to Δ*hly L. monocytogenes* and have found that, consistent with previous data, this strain fails to induce a robust response. These data are included for reference below. We are happy to include this figure as part of a supplementary figure, however, given that this has been repeatedly published we don't think it is necessary.

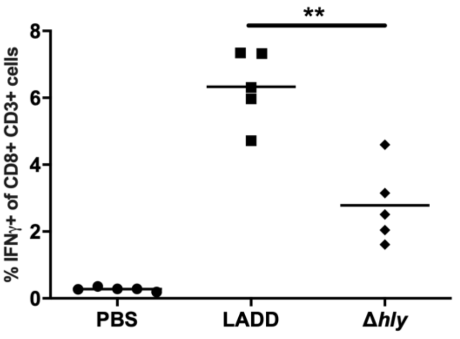

Furthermore, previous data demonstrated that Δ*hly L. monocytogenes* infection suppresses T-cell priming associated with WT *L. monocytogenes* infection (Bahjat et al., PLoS Pathog., 2009). As such, we found the experiment suggested by this reviewer assessing PGE_2 production during coinfection of the two strains very intriguing. Per this reviewer's suggestion, we coinfected mice with wild-type and Δ*hly L. monocytogenes* and assessed splenic PGE_2 levels. Coinfection of the two strains led to striking reductions of PGE_2 compared to wild-type infection alone, suggesting that vacuole-constrained *L. monocytogenes* actively suppresses PGE_2 production. These data are now included as Figure 2C.

Though *ex vivo* there was a non-statistically significant amount of PGE_2 produced by macrophages over mock-infected controls during Δ*hly L. monocytogenes* infection, the amount generated was minimal and not recapitulated in BMDCs. We suspect that this PGE_2 could be driven by a small amount of vacuolar escape by Δ*hly L. monocytogenes.* Previous data demonstrated that Δ*hly L. monocytogenes* is still capable of growth in human epithelial cells

(Portnoy et al., J Exp Med, 1988) and are able to escape to the cytoplasm in a number of other murine and human cell lines due to the activity of phospholipase C (Marquis et al., Infection and Immunity, 1995 and Grundling et al., J Bacteriol., 2003). In order to more fully understand the role of cytosolic access in driving PGE_2 production in BMDMs we assessed both a kinetic time course as well as higher infectious dose of Δ*hly L. monocytogenes* (Supplemental figure 1D-E, see response to reviewer 1, major comment 1a-b). In brief, we found that Δ*hly L. monocytogenes* did not induce PGE_2 at earlier or later time points and induced significantly less PGE_2 *ex vivo* even when infected at an initial dose much higher than wild-type infection. Furthermore, we have also assessed PGE_2 production *in vivo* during infection with Δ*hly L. monocytogenes*. When we immunized mice with even higher doses of Δ*hly* than demonstrated in our original Figure 2 (up to 100-fold more, the LD50 of Δ*hly L. monocytogenes*), we still do not detect PGE_2. We believe these data further demonstrate the dependency on cytosolic access for optimal PGE_2 production. These data are shown below for your reference.

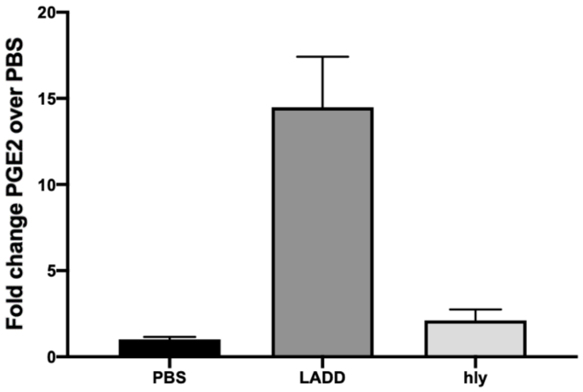

Additionally, though the hypothesis that impaired PGE_2 may be a reason why Δ*hly L. monocytogenes* induces worse T-cell responses is intriguing, we did not mean to imply that lack of PGE_2 was the sole reason why Δ*hly L. monocytogenes* induces suboptimal responses. We have softened the language to ensure we do not exclude other potential reasons why Δ*hly L. monocytogenes* fails to induce productive T-cell responses (eg. lines 156, 295-297, 443).

Finally, as suggested we assessed PGE_2 levels during inflammasome and type I IFN activation. We found that neither increases or decreases in type I IFN levels impacted PGE_2 production *ex vivo* (Supplemental figure 1F). In contrast, infection with a strain of *L. monocytogenes* (Lm-pyro) that hyperactivates the inflammasome and has been demonstrated to impair T-cell responses (Sauer et al., PNAS, 2011;Theisen et al., Infection and Immunity, 2016) surprisingly led to a slight decrease in PGE_2 levels. This may suggest that inflammasome activation impedes PGE_2 production or more simply that increased cell death from inflammasome activation led to decreased PGE_2 production. As discussed in response to reviewer 1 (comment 1e), we are currently preparing an additional manuscript assessing the role of inflammasomes and type I IFN on T-cell priming using novel caspase-1/11 and IFNAR knockout mice. A section of this manuscript includes a substantial analysis on eicosanoid levels during activation of these pathways. As such, we feel that further analysis is outside the scope of this current manuscript.

Reviewer #3: The responses of BMDM and BMDCs to Δhly is substantially different. In Fig 1B and 1C, BMDMs seem to be more responsive to Δhly than BMDC. Yet, the expression of COX2 is not decreased in PAM-challenged BMDM, but it is in BMDCs. Yet, in Fig 1D, the Δhly elicits decreased PGE2 production in both cell lines. What is the basis for such discrepancy?

We agree with the reviewer that this question is intriguing. Our current hypothesis is that the difference is PGE_2 is driven by other posttranslational mechanisms. For instance, terminal synthase activity can be modified by phosphorylation and/or presence of other cofactors such as ATP and glutathione. Similarly, COX-2 expression can be modified by a heme cofactor. Alterations in cofactor availability and/or posttranslational modifications would subsequently impact production of PGE_2. Perhaps in macrophages, for instance, expression of PGE_2 is controlled on the level of posttranslational modifications, whereas dendritic cells rely more so on protein translation. We have expanded upon this idea in the discussion (lines 551-557).

Fig.2 should be combined with Fig.1 or 3. It does not provide enough information to be a stand-alone fig. Also, a better rationale for Fig 2B should be provided.

We thank the reviewer for this suggestion. As PGE_2 production is completely eliminated during infection with vacuole-constrained strains *in vivo*, we feel that highlighting this in a separate figure is important. Furthermore, we performed an additional experiment assessing PGE_2 levels in mice infected with both wild-type and *Δhly L. monocytogenes* strain and found that coinfection led to abolished PGE_2 production (Figure 2C, see response to Reviewer 2, major comment 3). We believe that particularly given the addition of this additional panel, these experiments warrant an individual figure.

In terms of the rationale for Figure 2B, we felt it was necessary to quantify bacterial burden in order to ensure that the difference in signal was not due to enhanced bacterial clearance. *Δhly L. monocytogenes* does not survive as well *in vivo,* so we felt the rationale provided ensuring that the number of bacteria were equivalent was important.

In Fig.3, why the authors used the LADD strain instead of Δhly??? Using the LADD strain instead of Δhly is not a fair comparison with the experiments in Figs 1 and 2 and does not provide evidence about the role of cytosolic Listeria in PGE2 production. To help a non-T cell expert understand the data, a rationale for the investigation of CD8 producing IFN, rather than CD4, should be provided.

The LADD strain used in this study is attenuated in a way that still allows T-cell priming by accessing the cytosol (Brockstedt et al., PNAS, 2004). In contrast, *Δhly L. monocytogenes* fail to elicit robust T-cell responses (Bahjat et al., Infect. Immun., 2006, see response to reviewer 2 comment 3). Due to this, assessing changes in T-cell responses in our model to *Δhly L. monocytogenes* would not be productive, as no robust T-cell response would be generated. We have clarified this point in the text (lines 79, 155, 284-288, 535-536).

We choose to focus on $CD8^+$ T-cells in our analysis for two major reasons. First, though both $CD4^+$ and $CD8^+$ T-cells contribute to development of immunity to *L. monocytogenes*, $CD8^+$ T-

cells are the dominate cell type leading to protection and sterilizing immunity. Mice globally deficient in T-cells develop a chronic *L. monocytogenes* infection, highlighting the role of T-cells broadly in immunity (Bhardwaj et al., J Immunol., 1998). Importantly, however, studies relying on adoptive transfers or *in vivo* depletion show that $CD8^+$ T-cells are the most effective subset in mediating this protection in an antigen-dependent manner (Bhardwaj, et al., J Immunol., 1998; Harty et al., PNAS, 1992). Secondly, we chose to focus our analysis on $CD8^+$ T-cells specifically due to the development of *L. monocytogenes* as a cancer immunotherapeutic platform (Le et al., Semin Oncol., 2012). $CD8^+$ T-cells are a cell subset of interest in such therapies due to their potent antitumor capabilities. *L. monocytogenes* naturally induces a robust $CD8^+$ T-cell response, making it an optimal platform. For this translational benefit, we assessed how PGE_2 derived from different cell subsets influenced $CD8^+$ T-cells specifically. We assess $CD8^+$ T-cell responses both by quantifying number of antigen-specific cells generated (tetramer analysis) as well as by quantifying functionality. Functionality can be measured a number of different ways including cytotoxicity, granzyme expression, and cytokine production. For simplicity, we assessed functionality by cytokine production, measuring the percent and number of IFN-γ producing $CD8^+$ T cells. We have clarified the rationale for analysis of this subset in the text (lines 54-57, 335-338).

Fig 5 is indeed very interesting. However, panels with higher magnification and resolution should be provided.

We thank the reviewer for this comment and ensure that the final figure will be of quality resolution.

Part III – Minor Issues: Editorial and Data Presentation Modifications
Please use this section for editorial suggestions as well as relatively minor modifications of existing data that would enhance clarity.

Reviewer #1: I agree that there is now circumstantial evidence that phagocytes produce PGE2 and that PGE2 is necessary for full induction of CMI implying that the two are connected. However, I suggest that the authors change the title so as not to imply that they showed that phagocytes producing PGE2 were necessary for T-cell priming.

We have modified the title as suggested.

Reviewer #2:

1. Several references are not formatted properly. Lack page numbers, etc.

We apologize and have fixed the references.

2. Figure 2 shows that bacterial burdens of the delta-HLY strain are not lower in the liver, but PGE2 is measured in the spleens. The authors should include cfu data from the spleens as well.

Unfortunately, due to the labile nature of PGE_2 the spleens must be immediately flash frozen after harvest and therefore we have to use the entire spleen for eicosanoid analysis rather than use of a portion of the spleen to assess burden. However, we have also performed analysis at an even higher doses of Δ*hly* (up to 100-fold more, the LD50 of Δ*hly L. monocytogenes*) and still never are able to detect PGE_2, suggesting that difference is not due to burden (see data in response to reviewer 2, major comment 3).

3. Given the partial reduction in PGE2 and lack of impaired T response, it seems important to confirm efficiency of COX2 deletion in CX2FL/FL x CD11c-cre and/or Lyz2-cre mice. For example, by showing there is no COX2 protein and PGE2 production by phagocytes isolated from these mice. Perhaps this information will also be relevant in addressing some of the concerns above.

We agree that showing efficacy of deletion is important, particularly due to a lack of phenotype when assessing T-cell responses. We have included new immunohistochemistry data assessing COX-2 expression in the COX-$2^{fl/fl}$ CD11c-cre^{+} Lyz2-cre^{+} mice and see elimination of COX-2 expression in the PALS (Supplemental figure 6E, see response to Reviewer 1, major comment 2). The remaining COX-2 is in the marginal zone, as expected with inefficient gene deletion in marginal zone macrophages.

4. At least some of the control mice for the PGE2 experiments should be COX2FL/FL mice. Perhaps the flox itself impairs COX 2 expression and thus PGE2 irrespective of the CD11c or Lyz2 cre?

We apologize for the confusion – in our experiments we initially did our experiments using COX-$2^{fl/fl}$ cre^- mice as controls. For duplicates of these experiments we used either COX-$2^{fl/fl}$ cre^- mice or true wild-type (C57BL/6) mice. We see no differences in PGE_2 levels or T-cell responses between these two mice, suggesting that the insertion of the *loxP* site does not independently influence PGE_2 production. We have clarified this in the methods and in the figures (lines 634-639, figure labels).

5. I recommend the authors not use "Cd11c-cre" or "Lyz2-cre" as abbreviations for the cre x flox strains as this is confusing for the reader.

We thank the reviewer for the comment and have changed the nomenclature throughout the text.

Reviewer #3: Also, the absolute concentration of PGE2 should be shown instead of fold changes. This reviewer understands that transforming the values for fold change might be required due to intrinsic variations in concentration, but the raw numbers of the basal levels and challenge with WT bacterial should be mentioned in the fig. legends. Is the n=2 of the experiments? If so, it is not appropriate to perform statistical analysis in n=2

We agree that absolute concentrations would be interesting to include. However, mass spectrometry is incredibly sensitive and as such ion counts vary between biological replicates day-to-day. Furthermore, standard curves were not run in every replicate for every assessed eicosanoid. For these reasons, we have more confidence in a fold-change representation rather than absolute value. To clarify our analysis - we represent our data as a value normalized to deuterated PGE_2 (PGE_2-d_4) and fold-change or percent compared to either PBS or WT infection. Each sample upon harvest is spiked with a uniform amount of PGE_2-d_4 to assess extraction efficiency. Upon mass spectrometry analysis, samples with levels of PGE_2-d_4 at higher or lower than average values are normalized to ensure any differences in PGE_2 are not simply due to differences in extraction efficiency for that sample. These values are then compared to the average PGE_2 ion count for PBS or WT infected samples, as designated in the figures. In terms of replicates, our PGE_2 and T-cell experiments utilized 3-5 mice per group and was performed in biological replicate, allowing for use of statistics. We have clarified these methods in the manuscript (lines 719-722).

In the introduction (page 5, lines 92, 93), please change prostaglandin synthases to prostanoid synthases. Prostaglandin synthase only applies to PGE2 production.

We thank the reviewer for the correction and have changed the text accordingly.
